# A neuropathology-based approach to epilepsy surgery in brain tumors and proposal for a new terminology use for long-term epilepsy-associated brain tumors

**DOI:** 10.1007/s00401-014-1288-9

**Published:** 2014-05-25

**Authors:** Ingmar Blumcke, Eleonora Aronica, Horst Urbach, Andreas Alexopoulos, Jorge A. Gonzalez-Martinez

**Affiliations:** 1 Department of Neuropathology, University Hospital Erlangen, Schwabachanlage 6, 91054 Erlangen, Germany; 2 Department of (Neuro)Pathology, Academic Medisch Centrum (AMC), Swammerdam Institute for Life Sciences, Center for Neuroscience, University of Amsterdam, Amsterdam, The Netherlands; 3 SEIN, Stichting Epilepsie Instellingen Nederland, Heemstede, The Netherlands; 4 Department of Neuroradiology, University Hospital Freiburg, Freiburg, Germany; 5 Epilepsy Center, Neurological Institute, Cleveland Clinic, Cleveland, OH USA

## Abstract

Every fourth patient submitted to epilepsy surgery suffers from a brain tumor. Microscopically, these neoplasms present with a wide-ranging spectrum of glial or glio-neuronal tumor subtypes. Gangliogliomas (GG) and dysembryoplastic neuroepithelial tumors (DNTs) are the most frequently recognized entities accounting for 65 % of 1,551 tumors collected at the European Epilepsy Brain Bank (*n* = 5,842 epilepsy surgery samples). These tumors often present with early seizure onset at a mean age of 16.5 years, with 77 % of neoplasms affecting the temporal lobe. Relapse and malignant progression are rare events in this particular group of brain tumors. Surgical resection should be regarded, therefore, also as important treatment strategy to prevent epilepsy progression as well as seizure- and medication-related comorbidities. The characteristic clinical presentation and broad histopathological spectrum of these highly epileptogenic brain tumors will herein be classified as “long-term epilepsy associated tumors—LEATs”. LEATs differ from most other brain tumors by early onset of spontaneous seizures, and conceptually are regarded as developmental tumors to explain their pleomorphic microscopic appearance and frequent association with Focal Cortical Dysplasia Type IIIb. However, the broad neuropathologic spectrum and lack of reliable histopathological signatures make these tumors difficult to classify using the WHO system of brain tumors. As another consequence from poor agreement in published LEAT series, molecular diagnostic data remain ambiguous. Availability of surgical tissue specimens from patients which have been well characterized during their presurgical evaluation should open the possibility to systematically address the origin and epileptogenicity of LEATs, and will be further discussed herein. As a conclusion, the authors propose a novel A–B–C terminology of epileptogenic brain tumors (“epileptomas”) which hopefully promote the discussion between neuropathologists, neurooncologists and epileptologists. It must be our future mission to achieve international consensus for the clinico-pathological classification of LEATs that would also involve World Health Organization (WHO) and the International League against Epilepsy (ILAE).

## Long-term epilepsy-associated brain tumors (LEATs): what’s old, new, and blue?

**Fig. 1:**
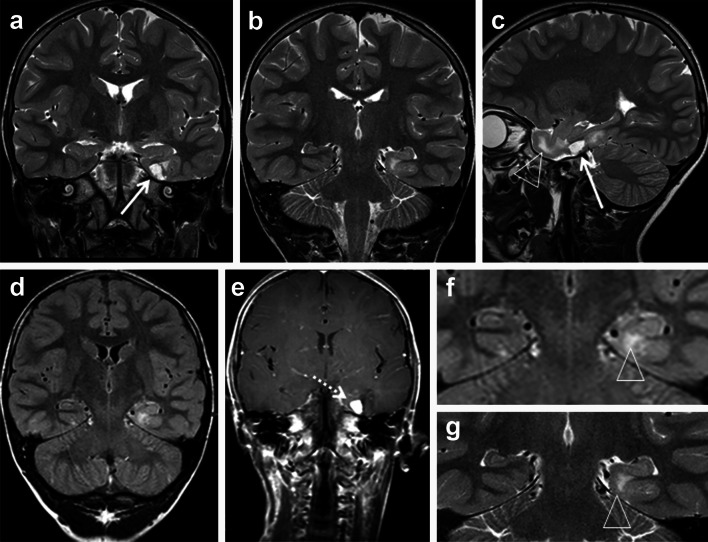
MRI characteristics of LEAT (CD34-positive BNET). **a**–**g** 6-year-old boy with drug-resistant temporal lobe epilepsy. Coronal T2-weighted (**a**, **b**), sagittal T2-weighted (**c**), coronal FLAIR (**d**), and coronal T1-weighted contrast-enhanced images (**e**) show a cortical/subcortical tumor with a cortical cyst (**a**, **c**: *arrow*), a contrast-enhancing nodule (*arrow* in **e**), and a T2-/FLAIR hyperintense white matter portion (**a**, **f**, **g**: *arrowhead*). Histopathological diagnosis was CD34-positive ganglioglioma (BNET)

**Fig. 2:**
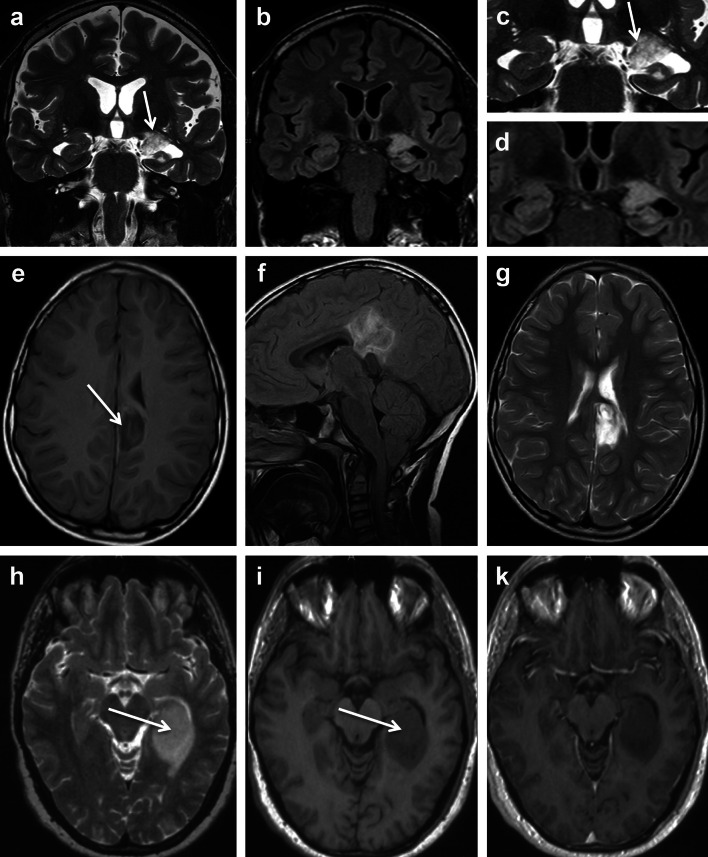
MRI characteristics of LEAT (DNET, ANET and INET). **a–d** 36-year-old man with temporal lobe seizures and histopathological diagnosis of DNET in the left amygdala and hippocampal head. The tumor revealed multiple tiny cysts, which can be resolved only by high-resolution T2-weighted (**a**, **c**: *arrow*), but not on FLAIR images (**b**, **d**). Axial T1-weighted (**e**), sagittal FLAIR (**f**), and axial T2-weighted images (**g**) showed a circumscript cortical and subcortical tumor at the base of the left parietal lobe dorsal to the cingulate gyrus. A ribbon-like cortical T1-hyperintensity (*arrow* in **e**) can be identified, and histopathology confirmed an ANET. **h–k** Isomorphic variant of astrocytoma (INET). The hippocampal and parahippocampal lesion has a space-occupying effect and a homogenous signal increase on T2 (**h**: *arrow*) and signal decrease on T1-weighted images (**i**: *arrow*), respectively. Signal changes suggested low cellularity with no contrast enhancement (**k**)

**Fig. 3:**
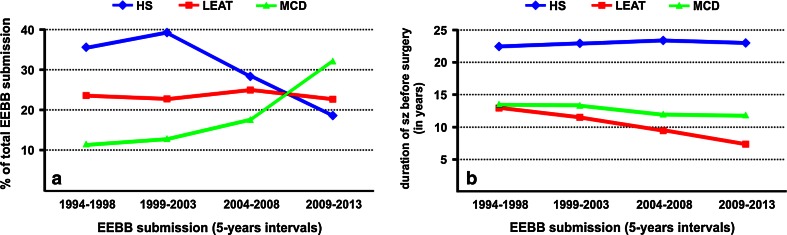
LEAT submission to the European Epilepsy Brain Bank (EEBB) in last 20 years. **a** During the last 20 years, submission frequencies have decreased for HS and increased for MCD (75 % FCD), whereas percentages of brain tumors associated with early epilepsy onset (LEAT) remained consistent over time. **b** Disease duration (time period from seizure onset until surgery; sz—seizures) has remained unchanged in HS and MCD during the last 20 years, whereas today’s patients with LEAT were operated 5 years earlier compared to mid 1990s (mean of 7.4 vs 12.9 years, respectively). EEBB submissions were grouped into four clusters of 5-year intervals between 1994 and 2013 to observe changes over time

Virtually any brain tumor can cause seizures. This review will focus on a particular group of brain tumors that usually manifest with seizure onset during early life (mean age = 16.5 years; Table 1), and which present with a broad histopathological spectrum of low-grade glial and glio-neuronal phenotypes [12, 42, 64, 66, 88, 94]. Herein, we designate this group as long-term epilepsy-associated brain tumors (LEATs or epileptomas). As in any other patient with a brain tumor, LEATs are readily detectable with standardized neuroimaging techniques (Figs. 1, 2). In contrast to most gliomas, slow LEAT growth and low risk for malignant progression may not necessitate immediate surgical intervention, nor will surgical strategies help to achieve long-term seizure control when aiming only at gross tumor resection. A common goal for successful treatment of these patients is the accurate identification of the epileptogenic zone, which may or may not match with the MRI visible lesion [31]. Advanced neurophysiological procedures including invasive EEG recordings may be needed in some patients [36, 43, 77], as discussed further below. However, weak agreement in the histopathological diagnosis of LEAT tumor entities [88] challenges any meaningful interpretation of published patient series. A major reason for poor agreement is the large spectrum of morphological variants when reviewing routinely stained hematoxylin and eosin (HE) sections, sharing one or more of the following features: (1) LEATs have a histologically variable appearance consisting of dysplastic neuronal and neoplastically transformed glial elements, mostly classified as glio-neuronal tumors by the World Health Organization (WHO) [42, 50]. (2) The vast majority of LEATs correspond to WHO Grade I [52, 53]. Reliable guidelines for the identification of tumors that carry a higher risk for recurrence and malignant progression are not available [12, 50]. (3) LEATs occur predominately in the temporal lobe and present with early seizure onset (with a mean age at seizure onset = 16.5 years). However, the average duration of epilepsy before surgical treatment adds another 12 years in our series of 1,551 patients (Table 1; Fig. 3b). (4) LEATs are likely to occur during brain development [8]. As such, tumors can be associated with Focal Cortical Dysplasia (FCD ILAE Type IIIb) [11, 58], or small tumor satellites infiltrating the adjacent neocortex (see chapters below). Which tumor entity preferentially follows which of these peculiar patterns, and whether any of these features contribute to enhanced epileptogenicity are important and yet unanswered questions in need of clarification. (5) LEATs do not share molecular features typically observed in diffusely infiltrating gliomas, such as IDH1 mutations or 1p/19q deletions [3, 57, 60, 95]. In contrast, the oncofetal marker protein CD34 can be frequently identified [7] and developmental genes are likely to be involved [40]. Mutations in B-RAF [45] or mammalian target of rapamycin (mTOR) signaling [5] have been also identified as key features in this group of tumors (see chapters below).

**Table 1:** Neuropathological findings in epilepsy surgery

Category	Numbers (%)	Age OP	Onset	Duration
HS	1,908 (32.7 %)	33.9 + 10.4	11.3 + 7.7	22.7 + 10.0
Dual	294 (5.0 %)	25.5 + 12.8	9.5 + 7.8	15.9 + 9.9
LEAT	1,551 (26.5 %)	27.9 + 12.3	16.5 + 10.1	11.8 + 8.8
MCD	930 (15.9 %)	18.2 + 12.0	5.9 + 5.7	12.3 + 9.1
Vascular	328 (5.6 %)	36.1 + 12.3	23.4 + 11.4	12.7 + 9.0
Glial scars	284 (4.9 %)	25.6 + 12.4	10.3 + 8.0	14.7 + 8.6
Encephalitis	96 (1.6 %)	20.4 + 12.6	13.3 + 9.4	8.2 + 7.1
No lesion	451 (7.7 %)	29.2 + 10.8	12.6 + 7.7	16.1 + 8.0
Total	5,842	28.6 + 12.5	12.4 + 8.9	16.5 + 10.1

Data retrieved from the European Epilepsy Brain Bank

*HS* hippocampal sclerosis, *Dual* dual pathology, *LEAT* long-term epilepsy-associated tumors, *MCD* malformations of cortical development, *Age OP* age of patients at surgery (in years), *Onset* age at onset of spontaneous seizure activity (in years), *Duration* duration of seizure disorder before surgical treatment (in years)

It is difficult to microscopically describe and histopathologically classify the broad spectrum of LEAT entities and their variants (Table 2) using standard eosin and hematoxylin (H&E) stains, which produce different histomorphological classification schemes of LEAT subtypes, i.e., the four different variants of a dysembryoplastic neuroepithelial tumor (DNT) with diffuse and simple forms, as well as complex and non-specific variants [13, 41, 89]. Other strategies have tried to incorporate rare or unusual subtypes into well-introduced WHO tumor entities, such as the GG [12]. As a matter of fact, meta-analysis of any published LEAT series is almost impossible and generates inconsistent results, despite adhering to the existing WHO classification system for brain tumors. In a previous review of eight international LEAT series (covering a total of 2,055 patients), we identified DNT and GG as the most prevalent entities [88]. However, the reported percentages for both categories showed a huge variance between published patient cohorts ranging from 7 to 70 %. These figures suggested a geographical bias, by which same tumors were classified either as GG or DNT, reflecting differences in neuropathology schools rather than reliable histopathological signatures. Again, the controversial and enigmatic question whether malignant progression can occur in a DNT (comprising any of its described variants) is a good, albeit unpleasant, example [21, 72].

**Table 2:** Neuropathological spectrum of brain tumors in an European epilepsy surgery series

Entity	Numbers (%)	Age OP	Onset	Duration
GG I°	673 (43.3 %)	24.9	12.8	12.7
GG II°/III°	77 (5.0 %)	26.9	14.2	11.0
DNET I°	256 (16.5 %)	25.2	14.7	10.7
PXA	38 (2.5 %)	29.3	18.8	12.2
INET	29 (1.9 %)	27.9	14.4	17.7
SEGA	16 (1.0 %)	20.1	12.3	9.0
ANET	5 (0.3 %)	19.7	2.0	13.0
ASTRO II°/III°	110 (7.1 %)	36.2	29.5	6.7
OLIGO II°/III°	97 (6.3 %)	38.6	24.5	12.5
PA I°	81 (5.2 %)	25.1	14.8	12.1
CYSTS	31 (2.0 %)	32.4	21.7	11.6
MENINGIOMA	26 (1.7 %)	46.5	38.9	8.4
NOS^$^	62 (3.2 %)	29.2	16.1	13.3
OTHER^§^	50 (4.0 %)	31.5	25.0	11.3
Total	1,551	27.9	16.5	11.7

Summary of 1,551 LEAT diagnosis collected by the EEBB (total *n* = 5,842); 709 female and 821 male patients were included. Grading according to WHO I°, II° or III° [50]

Age at operation (mean in years); Age at epilepsy onset (mean in years); Epilepsy duration (mean in years)

*GG* gangliogliomas, *DNET* dysembryoplastic neuroepithelial tumors, *PXA* pleomorphic xantoastrocytomas, *INET* isomorphic astrocytoma variants (analogous to WHO I°; [9, 10, 80]), *SEGA* subependymal giant cell astrocytomas, *ANET* angiocentric gliomas, *ASTRO* astrocytoma variants, *OLIGO* oligodendrogliomas including mixed gliomas, *PA* pilocytic astrocytomas, *CYSTS* arachnoid, dermoid or epidermoid cysts, *NOS* highly differentiated neuroepithelial tumors (not otherwise specified), *Other* all other tumors at rare frequency (<1 %)

Increasing availability of surgical tumor specimens should open the possibility to better characterize the molecular signature of each LEAT variant along with their molecular pathogenesis and epileptogenic potential. Notwithstanding, such studies will require use of a reliable terminology and histopathological classification that can be reproduced by any other laboratory. Prospectively designed randomized controlled trials for LEAT treatment are necessary to provide class 1 evidence for any suggested biomarker and classification scheme, an important goal that has never been addressed or realized up to this day.

## Epidemiological findings and neuropathological classification of LEATs: a matter of ongoing debate

The benefit of tailored resection strategies in patients with drug-resistant temporal lobe epilepsy is also recognized for the treatment of LEAT patients [52, 53, 86]. The comprehensive database of the European Epilepsy Brain Bank (EEBB) currently includes 5,842 samples, of which one quarter is diagnosed as LEAT (Table 1). With mean epilepsy duration of 11.8 years (Table 1), many tumors appear to escape detection in patients with early seizure onset or are medically treated for a long period of time period before surgery is considered as “ultima ratio”. Due to the preferential localization of LEATs in the temporal lobe, the various consequences of long-term epilepsy on cognition as well as social development and behavior require careful consideration [35] and seem to have already shifted the attitude of many epileptologists, neurologists and neurosurgeons toward earlier surgical intervention (Fig. 3b).

Neuropathological examination and diagnosis rely on microscopical inspection of surgical brain specimens and follow the current WHO classification and grading scale (last revised in 2007) [50]. This classification scheme has proven useful for the prediction of the biological behavior of most gliomas and other CNS tumor entities [51]. However, the broad spectrum of LEATs and their variable histomorphological features are not fully reflected within the current WHO grading system. Our EEBB collection of 1,551 LEAT cases included more than 15 different tumor entities in 709 female and 821 male patients (Table 2). Consequently, it is important to emphasize that proper neuropathological evaluation should be obtained from experienced centers. The need for such expertise is mandatory, given the considerable variability of histopathological phenotypes, which might result in “over-interpretation” of tumor progression leading to erroneous use of more aggressive therapeutic measures, even though most LEATs tend to have a very modest clinical behavior in the long run without bold risk of recurrence or malignant transformation [53]. At the same time, some tumors with a histologically typical, “benign”, glio-neuronal phenotype have been reported to rapidly turn into malignancies [54], underscoring the need for reliable biomarkers that could be used to predict the biological behavior of each individual tumor.

## Lack of diagnostic agreement requires new concepts: proposal of the A–B–C terminology of epileptomas

Given the broad histopathological spectrum of LEATs and the contradictory results published in the literature [88], we need to pursue better definitions and use standardized parameters for the neuropathological diagnosis of tumors associated with early onset epilepsy (Epileptomas). Such a proposal would also help reinforce research strategies toward a better understanding of the underlying biological nature (i.e., molecular pathogenesis), risk of malignant progression (i.e., predictive biomarkers), their epileptogenic potential (including presence or absence of FCD), and would allow for meaningful comparisons among published research studies and clinical patient series (i.e., epidemiological measures). Notwithstanding, there are no prospective, randomized, controlled trials of LEATs inpatients with epilepsy that demonstrate any better interrater agreement and superior predictive value of an alternative classification scheme. On the other hand, neuropathologists have already gone through these long and controversial discussions and disagreements 20 years ago with regard to the classification of malignant gliomas or medulloblastomas. As a result, the neuropathology community has now attained major achievements in specifying useful molecular diagnostic approaches and targeted therapies in these brain tumors [33, 34, 38, 81, 83, 87]. It seems that we are 20 years behind, when it comes to LEATs and associated epilepsies, and urgently need to shift our attention toward developing and implementing a new, clinically applicable consensus terminology for the neuropathological diagnosis of LEAT.

**Fig. 4:**
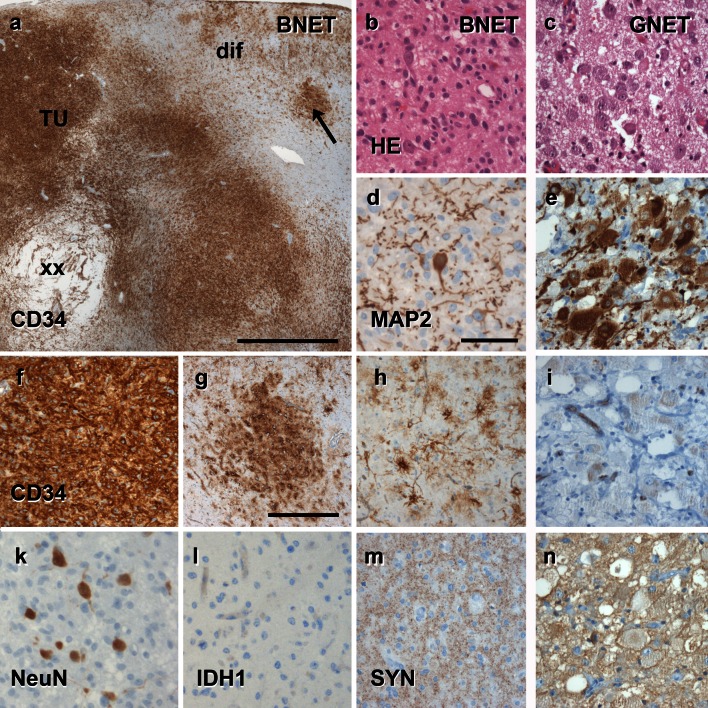
The spectrum of histomorphological and immunohistochemical hallmarks in LEATs with a glio-neuronal phenotype (A–B–C terminology). **a** BNET, immunoreactive for CD34 class II epitope (mAB QBend10, hematoxylin counterstaining; *first three columns*). Three different patterns can be distinguished. Pattern 1: (TU)—the bulk tumor is densely stained for CD34 (magnification in **f**). Pattern 2: clusters of tumor cells are visible in adjacent cortical areas (arrow, magnification in **g**). Pattern 3: diffusely infiltrating CD34-positive cells can be found in distant remote areas from the bulk tumor (magnification in **h**). Note, that patterns 2 and 3 may be interpreted as FCD ILAE Type IIIb when not using CD34 immunohistochemistry. **b, c** Routine histology stainings (H&E) reveal a biphasic pattern in BNET and a predominant gangliocytic patter in GNET. **d, e** Immunohistochemistry for the embryonic MAP2 epitope (MAP2e) is helpful to identify the neuronal component. Compared to diffusely infiltrating gliomas, the glial component does not label for Map2e (see Figs. 5, 6). **i** no CD34 immunoreactivity is visible in GNETs. **k–n** Supplementary markers are helpful to distinguish LEAT entities. NeuN labels dysplastic neurons in BNETs (**k**). IDH1 staining is always recommended as it is detectable only in neoplastically transformed glial cells (diffuse astrocytomas AII and oligodendroglioma OII; see Figs. 5, 6), but not in BNET (l). Synaptophysin (SYN) may be helpful to visualize the neuronal component in BNET (**m**) and GNET (**n**). *Scale bar* in **a** 2 mm, **d** 50 µm applies also to **b**, **c**, **e**, **i**–**n**. *Scale bar* in **g** 200 µm, applies also to **f**–**h**

**Fig. 5:**
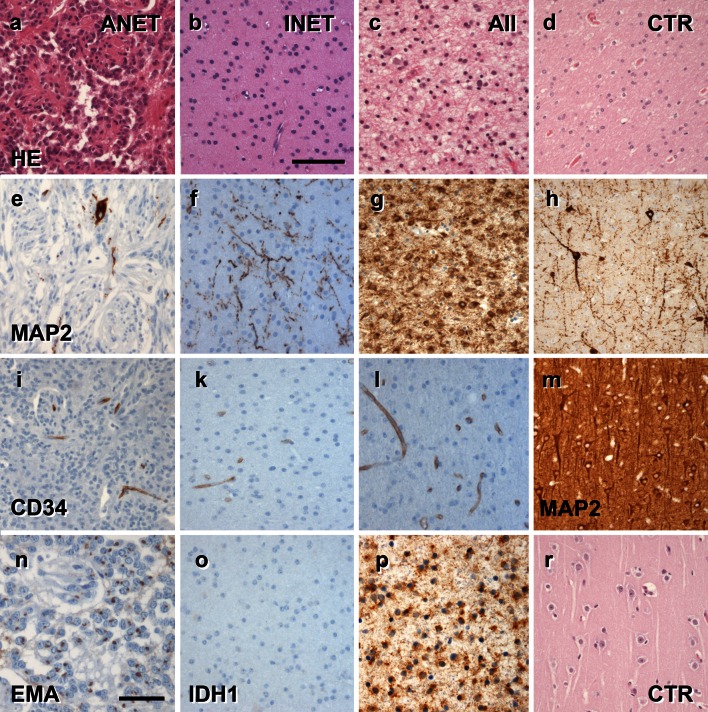
The spectrum of histomorphological and immunohistochemical hallmarks in LEATs with an astrocytic phenotype (A–B–C terminology). **a–c** Routine histology stainings reveal an angiocentric pattern in ANET (*first column*), or a prevailing astrocytic differentiation in INET (*second column*; similar to diffuse astrocytoma (AII; *third column*) shown in **c**). **d** HE staining from normal white matter (control/CTR) obtained from epilepsy surgery specimens; *forth column*. **e**–**h** Immunohistochemistry for the embryonic MAP2 epitope (MAP2e) is helpful to separate diffuse gliomas (AII, **g**) variants from any other LEAT entity (see also Figs. 4, 6). Only preexisting neurons and neuronal processes are visible in INET (**f**), comparable with heterotopic white matter neurons frequently seen in epilepsy surgery samples (**h**). Map2e-labeled cells in ANET (**e**) support the concept of a neuroepithelial tumor. **i–l** None of these variants shows CD34 immunoreactivity compared to BNET (see Fig. 4). **m** MAP2e staining in normal neocortex (layer 3; same specimen than **d**, **h**, and **r**). **n–r** Supplementary markers are helpful to distinguish LEAT entities. EMA dots can be frequently encountered in ANET variants. IDH1 staining is recommended to exclude neoplastically transformed glial cells in diffuse astrocytomas (**p**; see also Fig. 6), but not in INET (**o**). *Scale bar* in **b** 50 µm, applies to all images, with the exception of (**n**) 20 µm

**Fig. 6:**
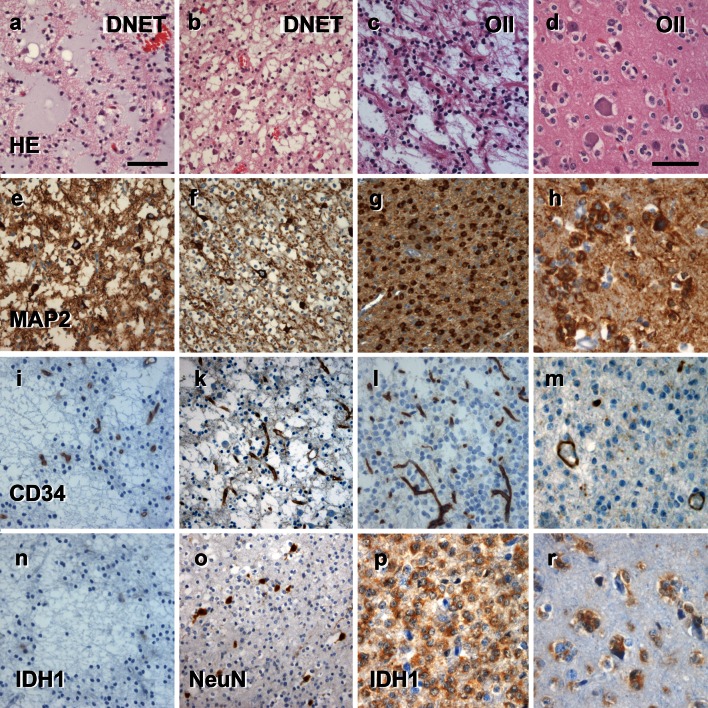
The spectrum of histomorphological and immunohistochemical hallmarks in LEATs with a clear cell morphology (A–B–C terminology). **a–c** Routine histology stainings reveal the specific glio-neuronal element in DNET (**a**, **b**; *first and second columns*), which may be difficult to distinguish from some oligodendroglioma variants (OII; *third and forth columns*). **d** OII infiltrating diffusely into neocortex (also in **h**, **m**, **r**). **e–h** Immunohistochemistry for the embryonic MAP2 epitope (MAP2e) is variable in DNET variants with abundant labeling of glial and neuronal cells (**e**) or neurons only (**f**). Neoplastically transformed glial cells always label with MAP2e in OII (**g**, **h**). **i–m** CD34 immunoreactivity is always devoid in DNETs as well as clear cell glioma variants. Immunoreactivity of vascular endothelium can be used as positive control. **n–r** Supplementary markers are helpful to distinguish LEAT entities. IDH1 staining is always recommended as it is detectable only in neoplastically transformed glial cells (oligodendroglioma OII, **p**–**r**), but not in DNET (**n**). NeuN may be helpful to visualize the neuronal component in DNETs (**o**). *Scale bar* in **a** 50 µm, applies to images **b**, **c**, **e**, **f**, **g**, **i**, **k**, **l**, **n**, **o**, **p**. *Scale bar* in **d** 20 µm, applies also to **h**, **m** and **r**

Given that the spectrum of histopathological changes of most glio-neuronal tumors is highly variable and cannot be sufficiently characterized by H&E staining alone, we recommend the introduction and routine use of a selected panel of immunohistochemical stainings that would allow neuropathologists to generate an appropriate differential diagnosis of LEATs as well as their distinction from other gliomas, i.e., CD34 [7, 8, 28, 61] and MAP2 [6, 10, 12, 76, 84] (Figs. 4, 5, 6). According to published experience with these immunohistochemical markers [53], we propose a terminology, which would allow us to classify the majority of tumors that fall under the entire LEAT spectrum. As an example, the frequently used term “Ganglioglioma” could be replaced by two new terms based on the presence or absence of the CD34 oncofetal class II epitope [7, 8, 28, 61]. The proposed terms are: basic (oncofetal) neuroepithelial tumor “BNET” with CD34 expression, compared to a (predominant) gangliocytic neuroepithelial tumor “GNET” without CD34 expression (Fig. 4). This new terminology would likely help avoid the confusion that characterizes previous reports, and which have included a broad histopathological spectrum of gangliogliomas and DNTs associated with various degrees of gangliocytic components [13, 66, 84, 88, 89]. BNETs will always be devoid of MAP2 labeling within their glial cell component (Fig. 4; Table 3). The MAP2 epitope of interest is an alternatively spliced transient (embryonic) isoform including exon 13 (MAP2E) [6, 82]. This isoform is likely to occur also in astroglial precursors [6] and can be detected in the vast majority of diffusely infiltrating gliomas [10, 76]. We consider this as important evidence for excluding any semi-malignant tumor mimicking LEAT variants, i.e., if only fragmented surgical specimens are available for diagnostic review and evidence for entrapped preexisting neuronal subpopulations is difficult to obtain. Mutation-specific antibodies directed against the IDH1 enzyme are important markers highly recognized in diffuse gliomas [3, 18, 19, 37, 60], and should be also encountered in the histopathologic LEAT work-up panel to identify and differentiate diffuse glioma variants.

**Table 3:** Terminology proposal for long-term epilepsy-associated tumors

WHO	ILAE^a^	CD34	MAP2_glial_	MAP2_neuronal_	IDH1
ANET^b^	ANET^b^	–	+/−	Preexisting	–
GG^c^	BNET^g^	+	+/−	Dysplastic	–
DNET^d^	CNET^d^	f.c.t.c.	f.c.t.c.	f.c.t.c.	–
DNET^e^	DNET^e^	–	+/−	Floating Neurons	–
–	ENET^g^	–	+/−	+/−	t.b.d.
GG^c^	GNET^g^	–	–	Dysplastic	–
A II^f^	INET^g^	–	–	Preexisting	–

CD34 (class II epitope) labels non-endothelial (oncofetal) cells [7]. MAP2 refers to the embryonic MAP2E isoform including exon13, which can be detected in neoplastically transformed glial cells [6, 85], in contrast to neurons that express the matured high-molecular weight MAP2

Astrocytomas and Oligodendrogliomas should always be excluded by testing for IDH1 mutations (as well as 1p/19q losses), which are considered as the most significant discriminators between astrocytomas/oligodendrogliomas and A–B–C LEATs. Pilocytic astrocytomas, pleomorphic xanthoastrocytomas, subependymal giant cell astrocytomas, papillary glio-neuronal tumors, or rosetted glio-neuronal tumors may also be identified in patients with chronic epilepsy (Table 2; Fig. 5) but show pathognomic histopathological, immunohistochemical and molecular features that help to distinguish from LEATs addressed by this new ABC terminology proposal

*f.c.t.c.* follows composite tumor components, *t.b.d.* to be determined, *ENET* some LEATs that do not express CD34 may still escape classification within this system (*ENET* epileptogenic neuroepithelial tumor—Epileptoma/NOS)

^a^Proposal from a Task Force of the ILAE Commission on Diagnostic Methods

^b^[47, 91]

^c^ [7, 9, 12, 53]

^d^[39, 67]

^e^[10, 25, 52, 80, 89]

^f^[6, 9]

^g^Proposed new A–B–C terminology for LEAT entities

The microscopic appearance of BNETs shares the broad spectrum of gangliogliomas described in the literature with dysplastic neuronal and neoplastically transformed glial components (Fig. 4) [5, 12]. BNETs will frequently reveal calcification, lymphocytic infiltrates and protein droplets. The dysplastic neuronal component may be difficult to identify when searching for multinucleated neurons, which requires additional immunohistochemical measures, i.e., synatophysin [29], NeuN [93] or MAP2 [10]. Few BNET variants can thereby present with a predominant glial cell population (astroglial or with clear cell morphology), which need to be distinguished from diffusely infiltrating astrocytomas or oligodendrogliomas using appropriate immunohistochemical markers, such as IDH1 and MAP2 [10, 18].

The growth pattern of BNETs may be nodular but diffuse infiltration into adjacent neocortex and white matter is also frequently encountered. This growth pattern becomes even more evident using CD34 immunohistochemistry, as the class II CD34 epitope is not expressed in the adult normal mammalian central nervous system or any other neuroimmunological or neurodegenerative disease. In BNETs, CD34 immunolabeling presents with three common patterns (Fig. 4). Type I labels bulk tumor cells (Fig. 4e), Type II diffusely infiltrating tumor cells (Fig. 4h) and Type III what we called “tumor satellites”. These are small clusters or nodules of CD34 immunoreactive cells in remote cortical areas. These may not be detectable by H&E staining, and often cause misinterpretation as FCD Type IIIb [11]. Such satellites can be immunohistochemically identified even if small or fragmented surgical specimens are available for histopathological review. However, described patterns for CD34 immunoreactivity may not be simultaneously present in each tumor specimen. Some BNET will reveal all three patterns, which usually require an anatomically well-preserved surgical specimen. On the other hand, BNETs may only show Type II or III patterns, in particular if fragmented specimens are available. However, their presence is sufficient to classify them as BNET. So far, no other disease condition or normal brain cell population has been shown to express the class II CD34 epitope. Unfortunately, the nature and origin of CD34 immunoreactive cells have not been clarified in BNETs, but most likely present neoplastically transformed neural precursor cells [7], although normal precursor cells or any other normal adult cell population in the human brain were never shown to express the CD34 class II epitope [7].

A challenge for the differential diagnosis is to distinguish BNETs from other brain tumors expressing CD34, such as pleomorphic xanthoastrocytomas [73]. BNETs may also share cytological features of pilocytic astrocytomas and oligodendrogliomas, which need careful consideration (Figs. 4, 5). Immunohistochemical stainings using IDH1 and MAP2 will help to clarify their differential diagnoses [10].

The term “DNET” and its current WHO definition will remain unchanged in this proposal. As originally described by Daumas-Duport, DNETs exhibit the characteristic multinodular appearance and specific glio-neuronal element as most prominent histopathological features [25]. These tumors should always be devoid of CD34 [10] and IDH1 immunoreactivity (Fig. 5). MAP2E can be present in the neuronal and glial component of DNETs. Diffuse or non-specific forms, characterized by a clear cell morphology, [13, 21, 22, 41, 89], are not encountered in the proposed A–B–C scheme. Such entities will be classified according to their CD34 immunoreactivity profile, i.e., most tumors with diffuse oligodendroglial-like clear cell components will express CD34 and should be encountered into the “BNET” category. Complex forms of DNETs are already recognized by the 2007 WHO classification system, and will now be subsumed into the group of composite neuroepithelial tumors, “CNETs”. They are characterized by the co-existence of at least two distinct LEAT entities [13, 67]. Histopathological diagnosis should always include specification of tumor variants, i.e., CNET (DNET/BNET) (DNET/GNET) or any other possible configuration. However, composite tumors containing semi-malignant components, such as PXA [62], should be graded according to the highest degree of malignancy, and not classified as CNET.

The term “ANET” is synonymous to the angiocentric glioma and was introduced into the WHO classification system in 2007 [16, 50]. The term “ANET” and its definition will remain unchanged, and reserved for those LEATs that are characterized by the peculiar growth pattern [47, 91], and ependymoma-like differentiation displaying intracellular perinuclear EMA-immunopositive dots [68, 91]. Up to now, there is no report of CD34 immunoreactivity or IDH1 mutations in patients with ANETs [69], which matches with our experience. However, our series of ANETs also comprise tumors with a distinct neuronal component as well as variable other cytological morphologies [2, 17, 55, 56, 68]. We prefer, therefore, to re-introduce the original proposal of Lellouch–Tubiana and describe them as “neuroepithelial” tumors rather than pure “gliomas”. This proposal will need also further consideration by the WHO panel and finally awaits molecular diagnostic confirmation [71].

We would like to propose two new A–B–C LEAT entities. The “INET” is an isomorphic neuroepithelial tumor previously described as isomorphic astrocytoma variant corresponding to WHO Grade I, which lacks any CD34 and glial MAP2 labeling and showed benign long-term outcome without recurrence or progression for more than 13 years [9, 80]. In addition to the initial description of six cases in 2004, the EEBB has collected 29 of these stereotypically composed low-grade tumors (Table 2; Fig. 6). Their histomorphological characteristic shares a monomorphous astroglial appearance with low cellular density. Nuclear atypia and mitosis are absent. The tumor matrix is fibrillary and reveals intense labeling with antibodies directed against GFAP. This tumor can infiltrate adjacent cortical and archicortical structures and is usually visible on MRI as non-contrast enhancing mass lesion with homogenous signal increase on T2- and signal decrease on T1-weighted images (Fig. 1).

Any other epileptogenic neuroepithelial tumor with early seizure onset that does not match the proposed A–B–C terminology will be provisionally classified as “ENET—Epileptoma/NOS”. These tumors will most frequently share the histomorphological H&E spectrum of BNET but do not express CD34. They also lack other distinguishing histopathological signatures for their classification described here as ANET, DNET, GNET or INET.

The current proposal did not address any criteria of atypia or anaplasia as it is meant to start an interdisciplinary discussion about strategies how to better define LEAT entities in the near future, i.e., upcoming WHO classification systems. Long-term follow-up studies, preferentially obtained from prospective observational multi-center trials, will be mandatory to validate the clinical benefits of such new definition, i.e., with respect to tumor recurrence, malignant progression and postsurgical seizure control. These data cannot be retrospectively obtained from our EEBB and will need careful reevaluation from submitting centers. In addition, large-scale molecular genetic studies should be carried out to clarify signatures distinguishing A–B–C tumors from any other brain tumor entity. As already emphasized, existing literature is not helpful due to the inconsistent use of histopathological LEAT terminology [88].

## Molecular diagnostic findings in LEATs

A promising approach to overcome the difficult distinction of LEAT variants will be to identify specific molecular signatures, which is also helpful to clarify their histogenesis and epileptogenicity. Several recent studies have been performed and will be briefly summarized. However, the distinction between GG and DNT was often not used with same or comparable definitions and terminology (as discussed above) and all data will be summarized, therefore, as LEAT instead of what have been published to represent specific GG or DNT characteristics.

Analysis of chromosomal copy number alterations (CNAs) in a cohort of 61 patients with LEAT (specified as GG without reference to CD34) revealed gains of chromosomes 7 and 5 as common aberrations, the last being more frequent in LEAT compared to astrocytomas WHO grade II [40]. More recently, copy number profiling of a large cohort of 131 LEAT confirmed the occurrence of gains of chromosomes 5 and 7 in both GG and DNT subtypes, detecting different patterns of chromosomal alterations, including also somatic intra- and/or interchromosomal chromothripsis (Prawabo et al. personal communication). These observations suggest that LEATs share similar molecular features despite their large spectrum of morphological variants. However, this conclusion needs to be confirmed using any new proposal for tumor terminology. Loss of heterozygosity (LOH) of chromosomal arms 1p and 19q has been repetitively recognized and confirmed as molecular hallmarks in oligodendrogliomas [74], but rarely reported in LEATs [30, 65, 88], representing an additional diagnostic feature in cases in which the small size of the biopsy raises differential diagnosis of oligodendrogliomas. We also strongly recommend the evaluation of IDH1 and IDH2 gene mutations as useful tool for the differential diagnosis between WHO I° LEAT and WHO II° gliomas. IDH1 mutations can be recognized using a mutation-specific antibody directed against the most common mutation site in WHO II° gliomas [3, 18, 19, 37, 44], but generally absent in LEAT [15, 30, 46, 65, 88].

The combined molecular analysis of IDH1/IDH2 and BRAF mutations may represent the most powerful diagnostic tool in the evaluation of LEAT. A mutation of the BRAF oncogene (V600E mutation) has been recently shown in different entities within the LEAT spectrum, including “GG”, “desmoplastic infantile gangliogliomas” and “DNT” [20, 24, 30, 45, 65, 79, 97]. The presence of BRAF V600E mutations can be evaluated by direct DNA sequencing and by BRAF V600E immunohistochemical detection. However, the low cellularity of these tumors and the frequent admixture of normal cell components may produce false-negative findings. Accordingly, immunohistochemical studies emphasize the importance of performing DNA sequencing from macrodissected tissue, or even to apply single-cell laser-capture microdissection [45, 65]. The identification of mechanisms underlying tumor development in BRAF wild-type LEAT cases represents a major challenge for future studies, which also requires a reliable terminology and histopathological classification of these tumor entities. Interestingly, evaluation of BRAF mutation in a large cohort of both pediatric and adult LEAT reveals a positive correlation with the oncofetal marker protein CD34 [65]. These studies also support the pathogenic role of BRAF in low-grade glial tumors arising in young age groups and including entities, such as pilocytic astrocytomas and pleomorphic xanthoastrocytoma [32, 79]. A remaining issue is represented by the possible prognostic value of the BRAF status on recurrence-free survival [24] and postoperative seizure outcome [65], which will also require further evaluation.

The search for additional mechanisms involved in LEAT development and epileptogenicity has identified the mTOR signaling pathway as promising candidate [49]. The mTOR pathway acts as key regulator of cell size and growth control, proliferation, differentiation and survival during brain development, and increasing evidence supports the role of the TOR pathway in a wide variety of neurological disorders including both MCD and brain tumors [1, 23, 26, 96]. Enhanced mTOR pathway activation has been reported in LEAT, such as GG and DNT [4, 14, 65, 78], suggesting a pathogenic link between these tumor entities and focal MCD, such as FCD ILAE Type IIb and cortical tubers in tuberous sclerosis complex (TSC), [23, 49]. Mutational analysis of TSC1 and TSC2 has been performed in LEAT, but failed to identify mutations [59]. A somatic mutation was reported in intron 32 of the TSC2 gene in glial cells of one LEAT patient, but not in dysplastic neurons [5]. Thus, additional efforts are necessary to clarify the mechanism underlying the deregulation of mTOR in LEAT.

Understanding the mechanisms that underlie epileptogenesis in LEAT is essential to develop effective treatment in young patients in which drug-resistant epilepsy critically affects their daily life. Over the last decades, several hypotheses have been put forward to explain epileptogenesis in LEAT patients. These studies suggested the involvement of both tumor-related factors (i.e., tumor size, tumor location, cellular composition), as well as of peritumoral changes (i.e., hypoxia and acidosis, ionic and enzymatic changes, deposition of hemosiderin; [27, 70, 90]). A better definition of the molecular biology of LEAT may not only aid the development of a more targeted treatment involving specific pathogenic pathways, but may also contribute to explain the epileptogenic nature of specific LEAT entities, such as GG (i.e., BNET, GNET) and DNT.

## The road to epilepsy surgery in patients with brain tumors

Clinical management of patients with epilepsy due to brain tumors exemplifies an important area of conflict between medical specialities, i.e., neurooncology and epileptology [48]. As a matter of fact, many brain tumors manifest with seizures and will not require attention by specialized epileptologists. Patients showing clinical symptoms for increased intracranial pressure with/without seizures will have brain imaging to localize a suspected brain tumor. Most centers will then move forward to neurosurgical resection to reduce mass effects and to obtain a written histopathological report guiding further treatment options. This applies for most adult or elderly patients suffering from rapid tumor growth in cortical or subcortical areas. But we need to consider the red flags, which require a different approach and careful neurophysiological attention. As prominent example, children or adolescents with a brain tumor manifesting in the temporal lobe should be carefully examined, as these neoplasms usually belong to the spectrum of LEAT and not to semi-malignant or malignant gliomas. The benign nature of most LEATs and their slow cell growth will not require surgical treatment or any adjuvant radiation or chemotherapy [75]. Instead, control of spontaneous seizures should be the primary treatment target to reduce the risk for progressive cognitive impairment or adverse effects of medication. This can be exemplified by a mean disease onset of LEATs at 16.5 years (Table 1) and temporal lobe location in 80 % of our LEAT series. These patients should be consulted by a comprehensive and multi-disciplinary epilepsy center. Specialized expertise in reading neuroradiology images may already help to clarify the underlying nature of a given LEAT (Figs. 1, 2). If medical treatment does not achieve sustained seizure control [63], surgical resection should be recommended as primary and in many instances also curative treatment option, although a controlled and randomized clinical trial was performed so far only for surgical treatment of drug-resistant temporal lobe epilepsy [92]. If epilepsy surgery is considered in patients suffering from brain tumors, we have to anticipate two distinct clinical scenarios: (1) Tumor is located in non-dominant and non-eloquent cortical and subcortical areas. We do not need invasive monitoring in this situation. A generous resection involving the tumoral area plus adjacent cortex can be performed, guided by intraoperative electrocorticography (ECoG) if available. (2) Tumor is located in or in close proximity to eloquent cortical and subcortical areas. As discussed above, the MRI-identified tumor may or may not be intrinsically epileptogenic and perilesional cortex may also contribute to the generation and early propagation of the epileptic seizures. In this clinical scenario where there is an anatomical proximity with functional areas in the brain, such as a dominant hippocampus, Brocca’s speech or prefrontal motor areas, the anatomical boundaries between the epileptogenic zone, the tumoral area and functional cortical and subcortical areas can be very poorly defined if based only on non-invasive data. In this particular situation, invasive monitoring is strongly recommended.

There is, however, no agreement regarding optimal surgical strategies, and studies comparing lesionectomy with more extensive resection of the presumed peritumoral epileptogenic zone are lacking. Using not only a tumoral, but also an epilepsy surgery-oriented strategy, LEAT-associated epilepsies usually have excellent seizure outcome following appropriate surgical resection. Timing of surgical treatment is debatable in patients whose seizures are well controlled. When perceived benefits of surgical intervention are thought to overweigh any surgical or neurological risks, surgery can be offered early. In the decision making, one should also take into account the rare risk of tumor growth and malignant transformation, the real risk of later development of pharmaco-resistance, the potential side effects of long-term treatment with antiepileptic medications and the favorable outcome of surgical intervention, which of course depends on the location of the LEAT and its proximity to essential eloquent cortex.

The predilection of LEATs to involve the temporal lobe (and in particular the anterior, basal and mesial compartments of the temporal lobe) has direct implications in the presurgical evaluation and tailored surgical therapy, which may or may not include the hippocampus in the area of resection. LEATs involving or abutting the mesial temporal structures are usually associated with a more widespread epileptogenic network and should be regarded as a distinct electroclinical group. Moreover, LEATs can be associated with focal cortical dysplasia ILAE Type IIIb and, therefore, a “curative” lesionectomy has to be extended beyond the MRI-identifiable lesion. Tumor recurrence and malignant transformation are rather unusual, but should never be excluded, especially when dealing with certain histopathological subtypes [4]. In these instances, the focus of the neurology and oncology team is to not only limit tumor growth but also eliminate seizures, which can significantly impair quality of life. One cannot overemphasize the importance of appropriate surveillance plans following successful resection of such tumors. Lastly, controlled studies are needed to examine when it is appropriate to discontinue AEDs in patients with sustained seizure-freedom following surgical resection.

In conclusion, this review and our proposal of the A–B–C terminology are meant to initiate an interdisciplinary consensus discussion between neuropathologists, neurooncologists, epileptologists and any other medical and research discipline toward a better understanding of each tumors biologic behavior and successful medical treatment in patients suffering from drug-resistant focal epilepsy.
